# Seasonal and Environmental Influences on Microbial Community Dynamics in the Dunk River Watershed, Prince Edward Island, Canada

**DOI:** 10.1111/1462-2920.70469

**Published:** 2026-11-02

**Authors:** Sneha Surya Narayana Murthy, Jiacheng Chuan, Yefang Jiang, Wen Chen, Robert G. Beiko, Xiang Li

**Affiliations:** ^1^ Faculty of Computer Science and Institute for Comparative Genomics Dalhousie University Halifax Nova Scotia Canada; ^2^ Canadian Food Inspection Agency Charlottetown Lab Charlottetown Prince Edward Island Canada; ^3^ Research and Development Centre, Agriculture and Agri‐Food Canada Charlottetown Prince Edward Island Canada; ^4^ Ottawa Research and Development Centre, Agriculture and Agri‐Food Canada Ottawa Ontario Canada

**Keywords:** metagenomics, microbial ecology, riverine microbiome, seasonal dynamics, spatiotemporal dynamics

## Abstract

Riverine ecosystems are crucial biogeochemical hubs, with their microbial communities playing a key role in nutrient cycling and overall ecosystem health. This study used shotgun metagenomic sequencing to characterize the seasonal and environmental drivers of taxonomic and functional microbial community structure (read depth between 1.91 and 30.44 bp) in four tributaries of the Dunk River watershed in Prince Edward Island (PEI), a landscape shaped by intensive agriculture and groundwater‐dominated streamflow, where the seasonal and land‐use impacts on the river's microbiome remain understudied. Although taxonomic composition showed no significant spatial variation among sampling sites, functional profiling identified subtle but distinct spatial signatures, primarily driven at the site with the least human impacts. Mass effects driven by above‐ or below‐ground water dispersal pathways may override local selective pressures, resulting in a more uniform community structure across different locations. The study also identified significant correlations between certain rare microbial taxa and nutrient concentrations. This work provides critical baseline data on microbial ecology in PEI river systems, indicating that while temporal factors are the primary identifiable drivers among the measured variables, a substantial majority of the community variance remains unexplained, likely reflecting the inherent stochasticity of open riverine ecosystems.

## Graphical abstract

We characterized the taxonomic diversity of river microbial communities across diverse sites using high‐throughput sequencing. Our findings demonstrate that environmental drivers—from seasonal cycles to farm‐level inputs—predictably reshape the bacterial landscape, providing a framework for monitoring aquatic ecosystem dynamics.

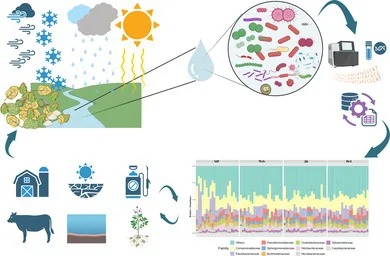

## 1 Introduction

River ecosystems are crucial biogeochemical hubs linking terrestrial and marine environments, supporting water supply, agriculture, and recreation (Gibson et al. 2018; Lu et al. 2023). Their ecological balance is driven by diverse microbial communities in the water and sediments (Boeraş et al. 2021; Liu et al. 2023; Taş et al. 2021). Riverine microbial communities exhibit significant temporal dynamics, often driven by seasonal changes in hydrology, temperature, and nutrient inputs. While microbial generation times can be short, substantial shifts in river conditions typically occur over seasonal cycles, impacting community structure and function (Gibson et al. 2018). Microbial diversity and composition can vary throughout the year: for instance, Kaevska et al. observed the highest microbial diversity in river water samples during autumn and early winter compared to other seasons (Kaevska et al. 2016). Liao et al. found that microbial community distribution patterns in the Chaobai River were dominated by denitrifying bacteria such as *Bacillus*, *Stenotrophomonas*, *Sphingobacterium*, and *Acinetobacter*, and were significantly influenced by environmental changes linked to seasonal shifts, particularly in warm temperatures (Liao et al. 2018). These seasonal fluctuations can influence not only overall community structure but also the relative abundance of specific taxa linked to environmental parameters (Dong et al. 2023; Zhang et al. 2023). Such temporal dynamics underscore the importance of considering seasonality when assessing river ecosystem health and microbial function (Kaevska et al. 2016; Liao et al. 2018). Studies have also linked seasonal events, like livestock grazing periods, to the conditional abundance of specific bacterial groups like Bacterioidales (Flynn et al. 2016), highlighting how seasonal human activities can intersect with natural cycles.

In tandem with season‐related shifts, specific environmental conditions can shape riverine microbial communities, including salinity, temperature, pH, dissolved oxygen, nutrients, organic carbon, and the sources of stream water (Doherty et al. 2017; Liao et al. 2018; Mason et al. 2016). Nutrient concentrations play a crucial role in regulating microbial growth, as their availability influences microbial metabolism and community structure (Hu et al. 2017; Zhang et al. 2021). Similarly, salinity gradients, particularly in estuaries or tidally influenced rivers, exert strong selective pressures, defining distinct microbial habitats and influencing metabolic processes like osmoregulation and nutrient cycling (Lozupone and Knight 2007; Tee et al. 2021; Thompson et al. 2017). The presence of heavy metals tends to favour low‐diversity communities that are highly adapted to stress (Chen et al. 2022; Tyson et al. 2004). Further, several correlations exist between bacterial taxa and environmental variables; for example, (Kaevska et al. 2016) Kaevska et al. (2016) found Actinobacteria (now Actinomycetota) concentrations that were negatively correlated with phosphorus and sulphate levels. Conversely, the study reported a positive correlation between Proteobacteria (now Pseudomonadota) and both sulphate and dissolved oxygen. Riverine water originates from surface runoff, groundwater discharge (i.e., baseflow), and wastewater inputs (Te Chow et al. 1988). These sources contribute different flow rates and distinct chemical and physical constituents at different times. Understanding their relative contributions and collective influence is vital for interpreting microbial dynamics.

Microbes also drive the cycling of key nutrients including carbon, nitrogen, phosphorus, and sulfur through processes like nitrification, denitrification, phosphorus absorption, and carbon fixation, with significant impacts on water quality (Balmonte et al. 2016; Battin et al. 2008; Boeraş et al. 2021; Gao et al. 2021; Hu et al. 2023; Wang et al. 2018; Zeglin 2015). Genera like *Pseudomonas*, *Acinetobacter*, and *Microbacterium* are involved in nitrate and phosphate removal (Banerjee et al. 2018; Chen et al. 2024; Ding et al. 2023). Anthropogenic nitrogen impacts these cycles, potentially leading to eutrophication and altered microbial communities (Burgin and Hamilton 2007; Fortin et al. 2021; Li et al. 2021; Wei et al. 2023; Zhang et al. 2021). Microorganisms are also primary decomposers, releasing nutrients and processing carbon (Battin et al. 2008; Fang et al. 2023; Zeglin 2015) while human activities have also been shown to impact decomposition rates (Liao et al. 2018).

Metagenomic sequencing is a cultivation‐independent molecular technique that involves random “shotgun” DNA sequencing of all genetic material from a sample. This approach has revolutionized river ecology research by overcoming cultivation biases and enabling comprehensive analysis of microbial composition and function (Eren and Banfield 2024; Grossart et al. 2020; Nam et al. 2023; Stewart 2012; Zheng et al. 2024). Given this context, taxonomic and functional profiling of these riverine microbial communities is a crucial element in understanding the impacts of environmental change (MacDonald et al. 2012). Further, metagenomic analysis has revealed intricate microbe‐environment relationships that have aided the understanding of riverine microbiomes (Bagagnan et al. 2024; Dong et al. 2023; Hopson et al. 2020; Huang et al. 2021; Nam et al. 2023; Pérez‐Cobas et al. 2020; Rodríguez‐Ramos et al. 2023; Zhang et al. 2023). An important consideration in shotgun metagenomics is sequencing depth, which directly influences taxonomic resolution and functional pathway discovery. However, empirical benchmarking demonstrates that shallow‐to‐moderate sequencing depths of 0.5 to 10 million (Gweon et al. 2019; Hillmann et al. 2018) reads per sample yield taxonomic and core functional community profiles with statistical support comparable to datasets generated at 100 million reads (Pereira‐Marques et al. 2024; Sunagawa et al. 2015) or more.

Extensive research indicates that temporal factors, particularly seasonality and hydrological fluctuations, frequently exert a stronger influence on aquatic microbiome structure than spatial variation (Fang et al. 2023). While spatial differentiation typically arises from localized environmental gradients or distinct land‐use inputs (She et al. 2024), the continuous downstream transport of water and high temporal variability in temperature and discharge often override these local spatial signatures, leading to temporal dominance (Gao et al. 2021). Despite this established understanding, a critical knowledge gap remains regarding how this temporal–spatial balance operates in groundwater‐dominated river systems.

The present study addresses this gap by investigating riverine microbiomes in PEI, Canada, a landscape characterized by intensive agricultural activities and a unique underlying hydrogeology consisting of a fractured Carbo‐Permian sandstone bedrock aquifer (Knysh et al. 2016). This highly permeable aquifer provides massive groundwater discharge to the surface, sustaining the streams almost entirely through stable baseflow during summer months (Jiang et al. 2015). It remains to be determined whether this continuous, geochemically stable groundwater input further homogenizes spatial diversity or if intense, localized agricultural nutrient loading creates overriding spatial niches.

While existing research in nearby Canadian maritime watersheds has begun to explore microbial dynamics in the context of broader ecosystem health (Bergbusch et al. 2025; Crossley et al. 2025; Deb et al. 2025), it does not account for the specific geochemical and hydrological conditions present on PEI. The island's unique environmental pressures, including high nutrient inputs from agriculture (Jiang et al. 2025) and recent climate‐related events like increased storm‐driven runoff (Jiang et al. 2015), could leave a distinct signature on its microbial communities. Therefore, this study aims to partition the relative contributions of seasonal, interannual, and spatial drivers on both the taxonomic and functional profiles of the riverine microbiome, determining how these unique hydrogeological constraints interact with established temporal patterns and establishing a critical baseline for this ecosystem.

Our specific objective was to investigate the seasonal, intra‐ and inter‐annual, and spatial differences that structure both microbial communities and their functional potential. Our study focused on the Dunk River, a river in central PEI that drains approximately 143 km^2^, approximately 80% of which is under agricultural production of potatoes, cereals, and pasture (Jiang et al. 2025). We hypothesize that temporal factors impact the microbial community structure and functional potential (Guan et al. 2023; Li et al. 2025; Wang et al. 2025). Spatial differentiation could be limited due to the underlying hydrogeology of the study area (Putman et al. 2021; Wang et al. 2025; Zhang et al. 2025), where a uniform, fractured bedrock aquifer discharges a consistent source of water, along with a minor influence of homogenizing dispersal. This hypothesis is also supported by the short residence time of water in the headwater areas and low groundwater temperatures in this high‐altitude region, which limit microbial growth (Jiang et al. 2015; Jiang and Somers 2009; Wang et al. 2025). Consistent with our hypotheses, we found that temporal factors had the largest impact on microbial community structure and function, though functional analysis revealed minimal site‐specific signatures previously obscured at the taxonomic level. Our results revealed predictable seasonal shifts, with community diversity decreasing during warmer summer months, and showed the relative abundance of key nutrient‐cycling pathways was consistently higher in 2020 compared to subsequent years.

## 2 Methods

### 2.1 Sample Collection & Processing

Water samples were collected from the outlets of the Breadalbane (Brd), Maple Plains (MP), Johnston River (JR), and Websters Creek (Web) tributaries within the Dunk River Watershed in PEI, Canada, for the years 2020 to 2022, covering the months from April until December (Figure 1, Figure S1b, Table 1). Sites were selected to varying degrees of anthropogenic pressure, such as farming intensity. This spatial design enables assessment of microbial community and functional shifts in response to seasonal changes in the freshwater ecosystem. Further, seasons were defined based on monthly sampling periods as follows: Spring (April–May), Summer (June–August), Fall (September–October), and Early winter (November–December). These categories reflect the regional climate cycle in Prince Edward Island (PEI) and were used to standardize temporal comparisons across sites and years.

**FIGURE 1:**
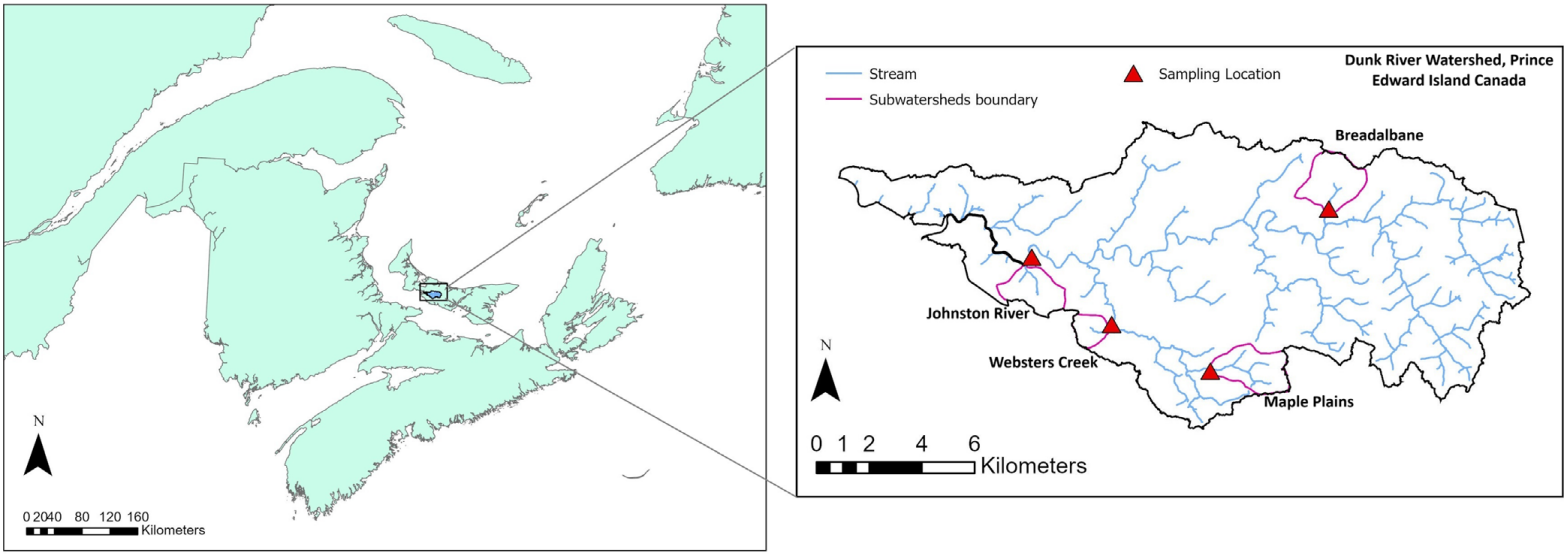
Study area and sampling sites across a river system reflecting spatial gradients. Water samples were collected from the outlets of the Breadalbane (Brd), Maple Plains (MP), Johnston River (JR), and Websters Creek (Web) tributaries within the Dunk River Estuary.

**TABLE 1:** Physicochemical and land use characteristics of sub‐watersheds sampled across the river system.

	Sub watershed	GPS coordinates (DMS)	Location description	Drainage area (km^2^)	Agricultural land (%)
MP	Maple plains	46° 18′ 19.5″ N, 63° 34′ 34.6″ W	Kinkora, PE C0B 1 N0	3.8	60
Web	Webster	46° 19′ 05.7″ N, 63° 37′ 54.5″ W	Lot 27, PE	1.3	95
JR	Johnston river	46° 20′ 25.4″ N, 63° 40′ 03.9″ W	Central Bedeque, PE C0B 1G0	3.0	86
Brd	Breadalbane	46° 21′ 29.2″ N, 63° 31′ 12.4″ W	Breadalbane, PE C0A 1E0	3.9	96

*Note:* Summary of GPS coordinates, drainage area, percentage of agricultural land use. Sample count distribution across sampling years (a), sites (b), seasons (c), and months (d), illustrating the temporal and spatial coverage of metagenomic samples is shown as S1b.

Because sampling occurred continuously from April through December, the collected water samples encompass a wide range of hydrological conditions. Samples from spring, fall, and early winter capture periods are heavily influenced by precipitation and surface runoff, whereas the summer samples capture strict baseflow conditions where streamflow is sustained almost entirely by groundwater discharge (Paradis et al. 2018).

At each sampling event, approximately 5 L of river water was collected in sterile, sealed plastic containers and transported to the laboratory under chilled conditions. Within 24 h of collection, water samples were sequentially filtered through a 1 μm glass fibre pre‐filter to remove particulate matter, followed by a 0.22 μm membrane filter (Sartorius Stedim Biotech, Germany) to capture microbial biomass. To provide experimental redundancy, 10 backup filters were processed from the same water sample at each site. However, only a single filter per site per time point was used for DNA extraction and downstream sequencing, resulting in an effective sample size of one per sampling event. All remaining unused filters were stored at −80°C.

Nitrate (NO_3_), chloride (Cl^−^), ammonium (NH_4_^+^), and total orthophosphate (PO_4_^3−^) were determined using a Lachat QuikChem 8500 flow injection analysis system (Lachat Instruments, Loveland, CO). These were monitored from 2020 to 2022 in the four selected streams. At the outlet of each stream, an insulated monitoring shed was installed, each housing an ISCO 6712 automatic sampler (ISCO Inc., Lincoln, Nebraska). The samplers at Maple Plains and Breadalbane were powered by onsite electricity, while the other two sites relied on solar power systems. Each sampler was programmed to collect a single 250 mL water sample per day. Samples were routinely transferred to 50 mL disposable centrifuge tubes and stored at 3°C for up to 1 week before analysis. While the automatic samplers collected daily records, only the records corresponding to the monthly field sampling timeline were considered in this study.

At each sampling site, a pressure transducer (Micro‐Diver, Van Essen Instruments, Delft, The Netherlands) was installed a few centimeters above the streambed to record stream stage and water temperature at hourly intervals. Monthly averages of stream temperature were then calculated to assess their influence on microbial diversity.

### 2.2 DNA Extraction & Quantification

DNA was extracted from the filters using the DNeasy PowerWater Kit (QIAGEN, Cat. No. 14900–50‐NF, Germany) according to the manufacturer's instructions. Following the initial filtration step, filter membranes were lifted from opposite edges using sterile forceps for DNA extraction. The homogenization method involved a 2‐step process, wherein Step 1 involved vortexing at maximum speed for 10 min, while Step 2 involved homogenization at 2000 RPM for 30 s, followed by a 30‐s pause. A second homogenization at 2000 RPM for another 30 s was performed, followed by the manufacturer's recommended sequence of washes using the standard buffers to ultimately elute the DNA with 60 μL of elution buffer.

The eluted DNA was then loaded into the 1% W/V agarose gel electrophoresis in the TBE buffer (pH 8.3) for verification for the quality and quantity. Gels were stained with ethidium bromide (MKCC7786, Sigma, USA) and imaged using the Bioanalytical Imaging System (Azure Biosystems Inc. USA). Capillary electrophoresis was used to further test the quality and quantity of DNA via QIAxcel Advanced (QIAGEN Hilden, Germany), with deionized water (ddH_2_O) serving as the negative control. In addition, the DNA concentration was measured three times using Qubit 3.0 Fluorometer (Thermo Fisher Scientific, USA) and the absorbance at 260 nm using anophotometerTM P300 (Implen, Germany).

### 2.3 Sequencing

DNA sequencing was performed at the CFIA Charlottetown laboratory. Next Generation sequencing (NGS) libraries were generated from the extracted DNA using the Nextera XT DNA Library Preparation Kit (Illumina, USA), and sequenced on the Illumina MiSeq platform (Illumina, USA) to generate paired end reads (300 bp × 2). FASTQ‐formatted raw metagenomic sequencing reads were quality checked using FastQC (Andrews 2010) supplemented with reports from MultiQC (Ewels et al. 2016) using default parameters. Preprocessing was performed using Atria v2.1.0 (Chuan et al. 2021) with default parameters to trim adapter sequences and low‐quality reads (Table S1).

### 2.4 Taxonomic Profiling

The trimmed reads were taxonomically assigned using Kraken2 v. 2.1.2 (Wood et al. 2019) with the standard database (RefSeq archaea, bacteria, viral, plasmid, human, UniVec_Core) using default parameters. After species annotation with Kraken2, Bracken (v2.7) (Lu et al. 2017; Wood et al. 2019) was used to predict the relative abundance of species in the samples, thus obtaining microbial abundances of streams at different taxonomic ranks. The TAXPASTA (TAXonomic Profile Aggregation and Standardization) merge v.0.7.0 (Beber et al. 2023) was used to summarize read counts across taxonomic ranks for multiple samples. Stacked bar plots were generated to visualize the relative abundance of microbial taxa at various ranks (phylum, family, genus, species). Data was prepared and plotted using R packages reshape (v0.8.10), ggplot2 (v3.5.2), viridis (v0.6.5), and stringr (v1.5.1), and were faceted by site, annotated with years, and sorted by month to highlight spatiotemporal trends. Any taxa with fewer than 10 reads were aggregated into an “Others” category.

### 2.5 Diversity Analysis

To account for variations in sequencing depth across the dataset and ensure strict comparability, taxonomic count tables derived from Bracken were rarefied to a uniform depth of 1,906,302 reads per sample (corresponding to the minimum library size) prior to alpha‐diversity estimation. Alpha‐diversity at the genus and species level was assessed using observed richness (the number of distinct taxa) and Shannon diversity, which accounts for both richness and evenness within the normalized library sizes. Shannon diversity (Ortiz‐Burgos 2016; Shannon 1948) was calculated as: H′ = −Σ (*pᵢ*…ln *pᵢ*), where *p*_*i*_ is the relative abundance of taxon *i*.

𝛽‐diversity was quantified to evaluate differences in community composition across samples and over time using Aitchison distance matrices computed from Bracken‐derived genus and species abundance profiles:

$$d_{\mathrm{jk}}=\sqrt{\sum_{i=1}^{n}{\left(\mathrm{CLR}\left(x_{\mathrm{ij}}\right)-\mathrm{CLR}\left(x_{\mathrm{ik}}\right)\right)}^{2}}$$

where *d*_*jk*_ is the distance between samples *j* and *k*, *x*_*ij*_ is the abundance of taxon *i* in sample *j*, and CLR(*x*) is the centered log‐ratio‐transformed value for sample *x*. Principal Coordinates Analysis (PCoA) was performed to visualize differences in community composition across sample types. Phyloseq (v1.48.0) (McMurdie and Holmes 2013), vegan (v2.6.6.1) (Dixon 2003), and mixOmics (v6.28.0) (Rohart et al. 2017) packages in *R* were used for this purpose.

### 2.6 Regularized Canonical Correlation Analysis

A multivariate statistical technique, regularized canonical correlation analysis (rCCA), was employed to examine the correlation between microbial abundance profiles and environmental factors (Legendre and Gauthier 2014). This multivariate technique reduces the data to fewer dimensions while keeping the strongest correlations between variables. The aim of the analysis was to uncover patterns of co‐variation, identify influential genera and environmental factors, and interpret the impact of nutrient dynamics on microbial composition. Variables were plotted within a unit circle based on their correlation with the selected canonical components, labeling the top three correlated genera based on each environmental variable.

Following the initial global analysis, an exploratory secondary rCCA was conducted restricting the dataset to summer samples. This subset analysis was pre‐specified based on the ecological hypothesis that summer baseflow conditions increase water residence time and strengthen deterministic taxa‐environment associations, which are otherwise obscured by stochastic hydrological transport during higher flow seasons. Given that PEI streams transition to nearly 100% groundwater‐fed baseflow during the summer (Mahmood et al. 2024), this period represents an ecologically constrained state free from the stochastic hydrological disturbances of other seasons.

Finally, to validate the rCCA‐derived correlation coefficients and ensure associations were not driven by rare taxa artefacts, we conducted a permutation‐based significance test (999 iterations). In each iteration, the rows of the environmental matrix were randomly shuffled to break the association between the microbial and environmental datasets, and the full rCCA model was refitted to build an empirical null distribution. Observed canonical cross‐loadings exceeding the 95th percentile of the absolute null distribution (permutation *p* < 0.05) were considered statistically significant.

### 2.7 Microbial Functional Profiling and Analysis

The functional potential of the microbial communities was characterized by analyzing trimmed sequencing reads using HUMAnN3 (Beghini et al. 2021). This software was used to identify and quantify genes, metabolic pathways, and functional modules. The analysis utilized the UniRef90 database, linked to species‐specific pangenomes from ChocoPhlAn, for sequence annotation. HUMAnN3 reported the relative abundance of detected gene families and biochemical pathways, providing a species‐level breakdown derived from MetaPhlAn3 (Beghini et al. 2021) results. The contributions of each species were estimated by summing their normalized abundances in Reads Per Kilobase (RPKs) for the pathways they were involved in. For cross‐sample comparisons, individual outputs were combined using the ‘humann_join_tables’ function. Identified gene families were organized into MetaCyc (Caspi et al. 2014) pathways, with a focus on those belonging to the MetaCyc categories of degradation/utilization/assimilation, focusing on those related to the cycling of nitrogen, phosphorus, sulfur, and carbon. The resulting data were then normalized to relative abundance. For all downstream analyses and visualizations, the aggregated pathway data encompassing contributions from all species (the non‐stratified table) was used. Because HUMAnN3's primary search tier relies on mapping against the pangenomes of species identified by MetaPhlAn3, a substantial majority of the resulting functional profile is implicitly shaped by the specific taxa detectable by MetaPhlAn3's marker‐gene approach (Beghini et al. 2021). Consequently, this functional taxonomic subset may differ from the broader community profile generated by Kraken2/Bracken. To prevent this discordance from affecting functional interpretations, we restricted all functional analyses to unstratified, community‐level pathway abundances and did not attribute specific metabolic functions to individual microbial taxa.

### 2.8 Multivariate Analysis and Statistical Testing

To investigate functional variation across samples, pathway abundance profiles were log‐transformed using the log1p function in *R* to stabilize variance. PCoA was then performed using the Euclidean distance metric to visualize differences in pathway composition among samples. The statistical significance of group‐wise differences in pathway profiles was assessed with a permutational multivariate analysis of variance (PERMANOVA) using the Adonis2 function from the Vegan (v2.6.6.1) R package (Dixon 2003), with 999 permutations. To further investigate the significant temporal effects identified by the global PERMANOVA, pairwise post hoc tests were performed using the pairwiseAdonis package in *R* (with 999 permutations). *p* values were adjusted for multiple comparisons using the False Discovery Rate (FDR) correction.

To assess the statistical significance of the regularized cross‐correlations between microbial taxa and environmental variables, a non‐parametric permutation test with 999 iterations was conducted. The rows of the environmental data matrix were randomly shuffled to establish an empirical null distribution and 95% confidence intervals for each taxon‐environment pair. This method evaluates the cross‐correlation matrix directly, avoiding the inflation of error rates common to canonical correlation permutations (Winkler et al. 2020). Observed cross‐correlations exceeding the 95th percentile of the absolute null distribution were considered statistically significant (*p* < 0.05).

### 2.9 Differential Pathway Abundance Analysis With MaAsLin2

Pathway abundances, originally quantified in RPK, were normalized to copies per million (CPM) using the humann_renorm_table function from HUMAnN3 (Beghini et al. 2021) to account for varying sequencing depths. These normalized profiles were analysed for associations with environmental variables using MaAsLin2 (Mallick et al. 2021) linear models. Separate models were constructed for Season (referencing Summer) and Year (referencing 2020). To capture a comprehensive view of shifts, directional pairwise contrasts were obtained by iteratively rotating the reference category. Significant associations were defined by a false discovery rate (FDR)‐adjusted *q* value < 0.05 and visualized using heatmaps. This directional approach results in asymmetric heatmaps as both coefficients and statistical significance are dependent on the specific reference level selected. This same framework was extended to the ‘Site’ variable to evaluate spatial heterogeneity across the watershed.

## 3 Results

### 3.1 Environmental Characteristics

To investigate temporal and spatial dynamics of the riverine microbiome in PEI, key environmental factors were first characterized across the study sites. Physical and chemical datasets included precipitation, biogeochemical elements, discharge, and water temperature, providing the context for microbial community patterns. Taxonomic and functional profiles were subsequently examined in relation to these environmental variables to assess linkages with overall ecosystem health. (Tables S2–S4).

#### 3.1.1 Precipitation and Stream Flow

Between 2020 and 2022, seasonal variation in rainfall directly influenced streamflow across the study sites (Table S2). In 2020, Summerside, PEI recorded only 238 mm of rainfall across the growing season months (April–August), substantially lower than the observed rainfall in both 2021 (389.6 mm) and 2022 (435.9 mm). The deficit was most pronounced in June (16.7 mm) and July (31.6 mm), which were far below the corresponding values in 2021 (40.0 mm and 132.5 mm) and 2022 (88.0 mm and 77.6 mm). Harrington, PEI showed the same pattern, with only 200.7 mm in 2020 compared to 472 mm in 2021 and 443.4 mm in 2022, driven largely by extremely low values in July (44.6 mm) and August (1.7 mm). August 2020 received just 1.7 mm compared to 29.5 mm in 2021 and 73.0 mm in 2022, while July 2020 (44.6 mm) was again much lower than 2021 (143.5 mm) and 2022 (99.8 mm). These reduced summer totals corresponded with diminished streamflow at Webster (Web), Johnston (JR), and Maple Plains (MP) in July–August 2020, whereas Breadalbane (Brd) displayed an anomalously higher July flow despite only moderate rainfall (44.6 mm at Harrington and 31.6 mm at Summerside).

#### 3.1.2 Water Temperature

Stream temperatures exhibited clear seasonal dynamics, with multi‐year mean values across sites rising from spring lows to summer peaks before declining in late autumn (Table S3). MP warmed most strongly, averaging ~17.5°C in July across years 2020–2022, before declining to ~6.8°C in December. Web peaked at ~16.1°C in July (2020–2022), while JR remained consistently cooler, with monthly means rarely exceeding 9.1°C. Brd was intermediate, reaching ~9.8°C in midsummer and dropping to ~7.3°C in December.

#### 3.1.3 Biogeochemical Elements

Autosampler measurements revealed distinct biogeochemical profiles among the four streams (Table S4). Web consistently exhibited the highest solute concentrations, with mean chloride at 91.2 mg/L, nitrate at 9.51 mg/L, and ammonium at 0.28 mg/L, while phosphate remained comparatively low (0.27 mg/L). MP was more dilute, with chloride (14.0 mg/L) and nitrate (1.92 mg/L) among the lowest across sites, but phosphate was the highest recorded (0.83 mg/L). JR showed low chloride (15.0 mg/L) and minimal phosphate (0.08 mg/L) yet maintained moderate nitrate levels (6.44 mg/L). Brd was intermediate, with chloride at 18.8 mg/L, nitrate at 9.09 mg/L, and elevated phosphate (0.79 mg/L). Overall, Web stood out for ionic and nitrogen enrichment, MP and Brd for phosphorus loading, and JR for moderate nitrate with otherwise low solute concentrations.

### 3.2 Sample Distribution and Read Statistics

A total of 87 metagenomic samples were distributed across the four defined time intervals: Spring (*n* = 15, April, May), Summer (*n* = 36, June, July, August), Fall (*n* = 24, September, October), and Early winter (*n* = 12, November, December) (Figure S1b). Quality‐control statistics derived from the processed metagenomic dataset indicate overall high sequencing quality and consistency across samples (Table S1; Figure S1a). Most samples exhibited sequence duplication rates below 5%, suggesting efficient library preparation. GC content was estimated to be distributed between 48% and 52%. On average, ~85%–90% of reads were retained after quality trimming. Read lengths predominantly ranged from 150 to 250 base pairs, reflecting consistent trimming and sequencing output.

Shotgun metagenomic sequencing of the environmental water samples yielded sequencing depths ranging from 1.91 million to 30.44 million reads per sample, with an overall mean depth of 7.19 million reads. To address library size heterogeneity and ensure rigorous comparability across the dataset, taxonomic count tables were sub‐sampled to a uniform depth of 1,906,302 reads prior to calculating alpha‐diversity metrics. The recalculation of Shannon diversity and Observed Richness at this uniform rarefied depth confirmed our initial diversity trends. Normalizing the data to a uniform depth demonstrated that the observed temporal and spatial variations in microbial community diversity are representative of ecological patterns rather than artifacts of unequal sequencing depth.

### 3.3 Riverine Microbiota Composition

Taxonomic profiling using Kraken2 and Bracken estimated an average of 1556 genera across all 87 samples, ranging between 957 and 1975 distinct genera (Table S5). An average of 5435 species were predicted, ranging between 3220 and 7675 species (Table S6). All samples were dominated by the phylum Pseudomonadota (68.43%), followed by Bacteroidota (13.88%) and Actinomycetota (11.84%) (Figure 2a). Comamonadaceae (20%), Flavobacteriaceae (10%), Pseudomonadaceae (5%), and Burkholderiaceae (4%), all from phylum Pseudomonadota, were the most‐abundant taxonomic families. The most abundant non‐Pseudomonata families were Nocardioidaceae (1.68%) and Streptomycetaceae (1.1%) from Actinomycetota, followed by Sphingobacteriaceae (0.88%) from Bacteroidota. Of the bacterial families detected, Comamonadaceae showed the highest concentration at the MP site, with a relative abundance of 23.15% (Figure S2a). The other locations had lower percentages for this family, with Web at 18.50%, JR at 19.58%, and Brd at 19.81%. Flavobacteriaceae followed a similar trend, peaking at the MP site with a relative abundance of 15.30%. In contrast, its presence was less pronounced at the other sites, measuring 7.96% at Web, 9.02% at JR, and 9.03% at Brd. Lastly, Burkholderiaceae was also most prominent at MP with 5.52%, while the remaining sites showed relatively lower levels, at 2.82% for Web, 3.24% for JR, and 2.80% for Brd.

**FIGURE 2:**
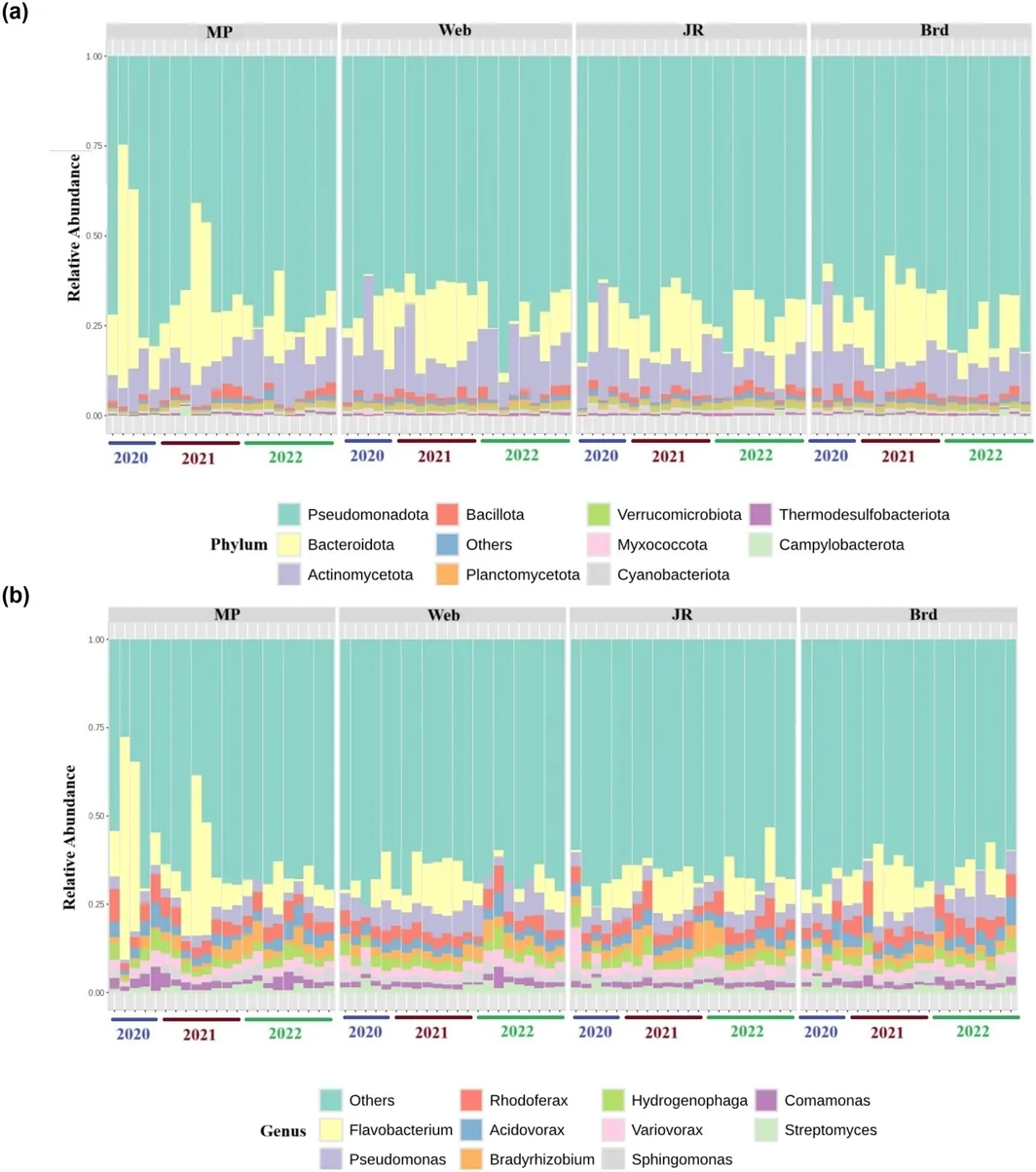
Taxonomic composition of different sites of river streams. Breadalbane (Brd), Maple Plains (MP), Johnston River (JR) and Websters Creek (Web). (a) Phylum level. (b) Genus level. The height of each bar corresponds to the relative abundance of the corresponding taxon at that site. Samples are shown in chronological order, with May to October sampled in 2020, May to December sampled in 2021, and April to November in 2022. Any taxa with a raw count of less than 10 reads are aggregated into the “Others” category.

Among the genera, *Flavobacterium* was the most abundant across all samples with an average abundance of 9.32%, followed by *Rhodoferax* (3.82%) and *Acidovorax* (3.22%) (Figure 2b). *Flavobacterium ammonigenes* and *Flavobacterium ammonificans* were identified as the predominant species in MP, with an average abundance of 8.14% and 4.35% respectively, as compared with only 0.10% and 0.18% for all other sites (Figure S2b). Further, these two species showed a decreasing trend across years, with average abundance of 19.98% (2020), 3.34% (2021), and 1.11% (2022) for *F. ammonigenes*, and 8.84% (2020), 3.33% (2021), and 0.89% (2022) for *F. ammonificans*.

### 3.4 Seasonal & Interannual Trends in Alpha Diversity

Alpha‐diversity patterns, calculated from the rarefied taxonomic count tables to control for library size variation, showed consistent seasonal fluctuations and interannual variability at all four sites (Figure 3). The calculation confirmed that sequence depth heterogeneity did not drive the observed ecological trends. Samples from 2020 showed slightly lower values (range = 4.56–5.11) compared to 2021 (5.06–5.41) and 2022 (4.62–5.45). In the summer season, 2020 (2.74–5.62) and 2021 (3.57–5.52) exhibited a broader range of diversity values with several low‐diversity outliers, as compared with 2022 (4.39–5.69) (Table S5).

**FIGURE 3:**
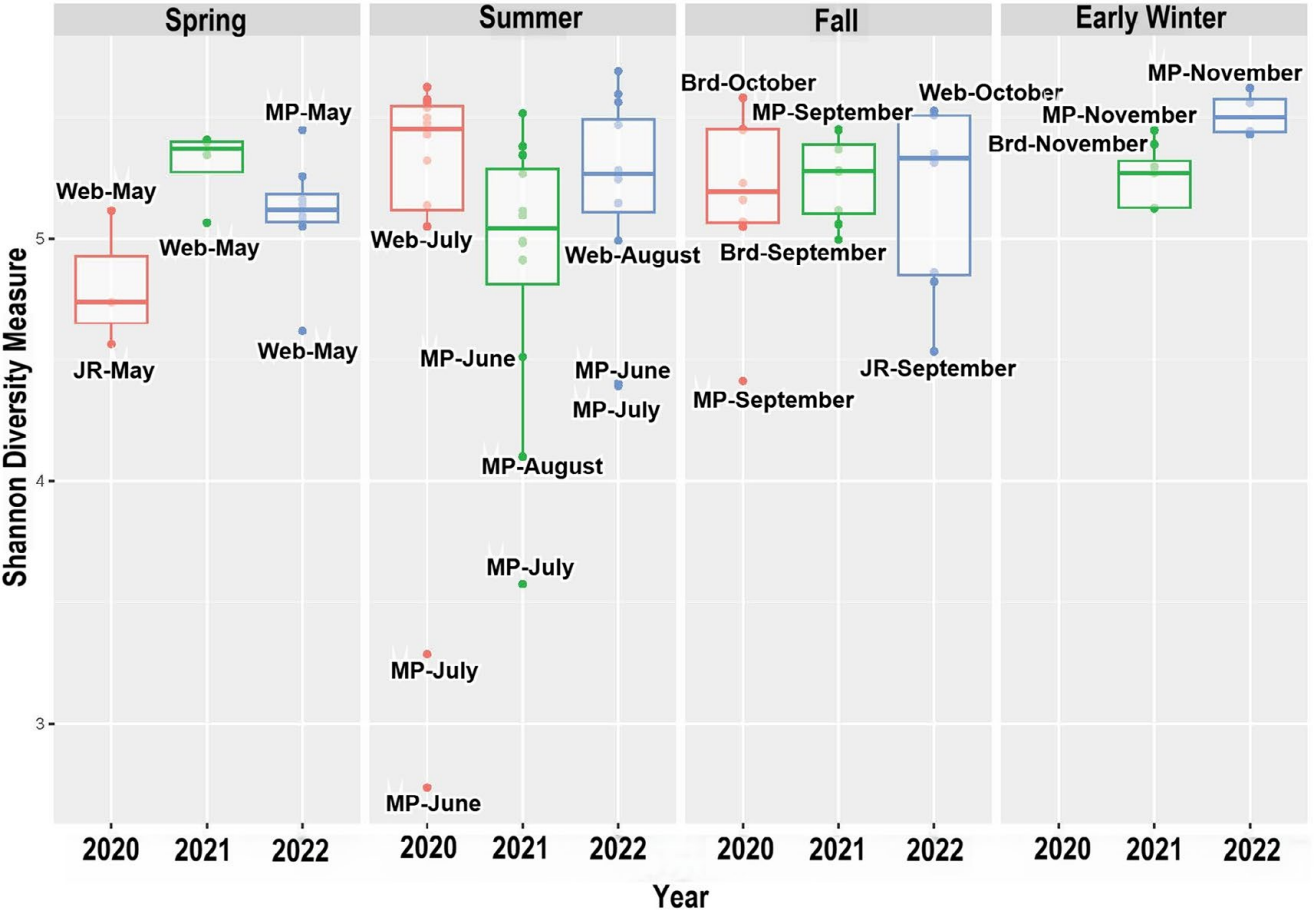
Seasonal and interannual trends in alpha diversity of riverine microbial communities. Boxplots depicting alpha‐diversity values across seasons and years. Each dot represents an individual sample. Outlier points are labeled with location and month.

Shannon diversity scores declined during the summer months, with the most pronounced reductions observed in 2020 and primarily associated with the MP site (ranging from 2.74 to 5.43). Across years and sites, summer samples exhibited both the lowest recorded values and a reduced mean index compared to other seasons (Figure 3, Table S5). In contrast, fall communities showed greater stability, displaying moderate variability in diversity across all 3 years. The strongest deviation was evident in the 2020 MP samples, where diversity consistently fell below levels observed at other sites and seasons. Early winter samples were consistently high in diversity (5.12–5.62), particularly in 2022 (5.43–5.62).

### 3.5 Temporal Dynamics and Taxonomic Structuring

Patterns of microbial community composition were assessed across seasons, months, years, and sites using Principal Coordinates Analysis (PCoA). While the overall effect size for taxonomic composition was small (PERMANOVA, *R* ^2^ = 0.05, *p* = 0.002), seasonal and monthly variation showed the strongest association with community structure. An examination of the PCoA plots showed that seasonal and monthly changes introduced some separation in the data, with samples from summer and early winter showing some clustering, while spring and fall samples largely overlapped. It is important to note that while the ellipses highlight general trends, there is substantial overlap between the groups, indicating a high degree of variation within seasons (taxonomic composition PERMANOVA, *R* ^2^ = 0.078, *p* = 0.002) (Figure 4a). Monthly clustering patterns also showed some separation, with early winter samples tending to form distinct clusters (taxonomic composition PERMANOVA, *R* ^2^ = 0.168, *p* = 0.001) (Figure 4c).

**FIGURE 4:**
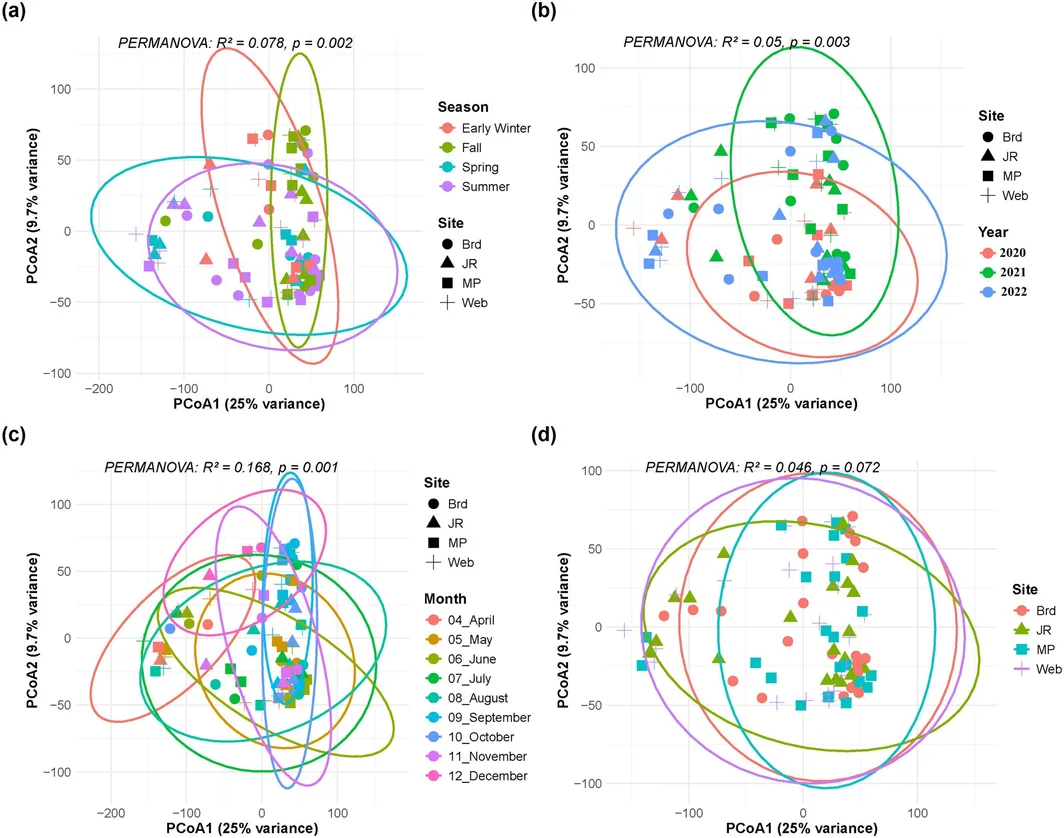
Principal coordinates analysis (PCoA) of riverine microbial taxonomic Profiles based on aitchison distance. PCoA plots illustrating differences in microbial community composition across (a) seasons, (b) years, (c) months, and (d) sites, based on Aitchison distance of taxonomic profiles obtained using Kraken2‐Bracken. Each point represents a sample, with shapes denoting year and colors denoting the grouping variable in each subplot. Ellipses indicate 95% confidence intervals for group centroids.

Year‐to‐year patterns were less pronounced than seasonal or monthly shifts, though some partial separation was observed between 2020 and 2022 samples (Figure 4b). A statistically significant overall effect of year was detected for taxonomic composition, albeit with a small effect size (PERMANOVA, *R* ^2^ = 0.050, *p* = 0.003). To determine if this represented consistent interannual variation or was driven by the anomalous low‐rainfall conditions of 2020, pairwise PERMANOVA post hoc tests with false discovery rate (FDR) correction were performed. No pairwise comparisons survived multiple testing corrections. The microbial communities in the non‐drought years were statistically indistinguishable from one another (2021 vs. 2022: *R* ^2^ = 0.050, *p* = 0.056), and 2020 did not differ significantly from 2021 (*R* ^2^ = 0.048, *p* = 0.056) or 2022 (*R* ^2^ = 0.017, *p* = 0.349).

In contrast, grouping by site showed substantial overlap of microbial community structures among river locations (Figure 4d). A small effect of site was detected, but the finding was not statistically significant for taxonomic composition (PERMANOVA, *R* ^2^ = 0.046, *p* = 0.072). It is important to note that the unbalanced sampling design across seasons may limit the statistical power required to resolve moderate spatial effects in this system.

“We utilized both seasonal and monthly temporal scales to evaluate community dynamics from a principled biological and statistical perspective. While seasonal aggregation provides a strong, ecologically intuitive framework for capturing broad macro‐climatic shifts, such as baseline thermal regimes, it can artificially smooth the data and obscure rapid community restructuring. Conversely, evaluating monthly variation provides the necessary temporal resolution to capture acute, transient hydrological disturbances that act as short‐term environmental filters. This dual approach proved both biologically and statistically advantageous, as our quantitative variance partitioning confirmed that monthly variation independently captured the highest proportion of community variance for both taxonomic (*R* ^2^ = 0.168) and functional (*R* ^2^ = 0.225) profiles, revealing finer‐scale transitional dynamics that are otherwise obscured when aggregated into seasons.”

### 3.6 Environmental Associations With Microbial Community Composition

We employed regularized Canonical Correlation Analysis (rCCA) to examine potential relationships between the river's microbial community composition and key environmental variables, specifically nutrient concentrations across a spatiotemporal gradient. The initial rCCA, conducted with all 87 samples, placed all microbial genera near the center of the unit circle, indicating a lack of substantial correlation with nitrate, chloride, phosphate, ammonium, water temperature, and regional rainfall (Figure S4). Because high water flow can introduce stochastic transport that masks biological signals, we hypothesized that reduced hydrological mixing and elevated temperatures during summer low flow conditions would enhance deterministic environmental filtering. To explore this, we performed an exploratory secondary rCCA restricted to summer samples to determine if taxa‐environment coupling becomes more detectable under these ecologically constrained baseflow states. Regional rainfall data was also incorporated into the model to assess the influence of precipitation; however, rainfall exhibited near‐zero canonical loadings and no significant associations with the microbial community across all canonical components.

The summer‐sample rCCA analysis highlighted distinct associations between microbial taxa and environmental variables (Figures 5 and S5). Temperature was the strongest correlate, with *Stigmatella* (*r* = 0.60), *Niveispirillum* (*r* = 0.58), and *Actinotignum* (*r* = 0.55) showing strong positive associations. Among the chemical parameters measured, chloride showed the strongest correlations, with *Botrimarina* (*r* = 0.59), *Halioglobus* (*r* = 0.50), and *Kineobactrum* (*r* = 0.48) showing the strongest associations. Nitrate correlations were lower, with *Botrimarina* (*r* = 0.33), *Halioglobus* (*r* = 0.30), and *Kineobactrum* (*r* = 0.28) again showing the strongest correlations. Ammonium and phosphate showed very low initial correlations with the microbial community. Subsequent 999‐iteration permutation testing confirmed that associations with phosphate and ammonium were not statistically significant for any of the tested taxa. Consequently, spurious initial associations involving rare groups, such as the viral genus *Megavirus*, were flagged as likely classification artifacts and excluded from the validated canonical results.

**FIGURE 5:**
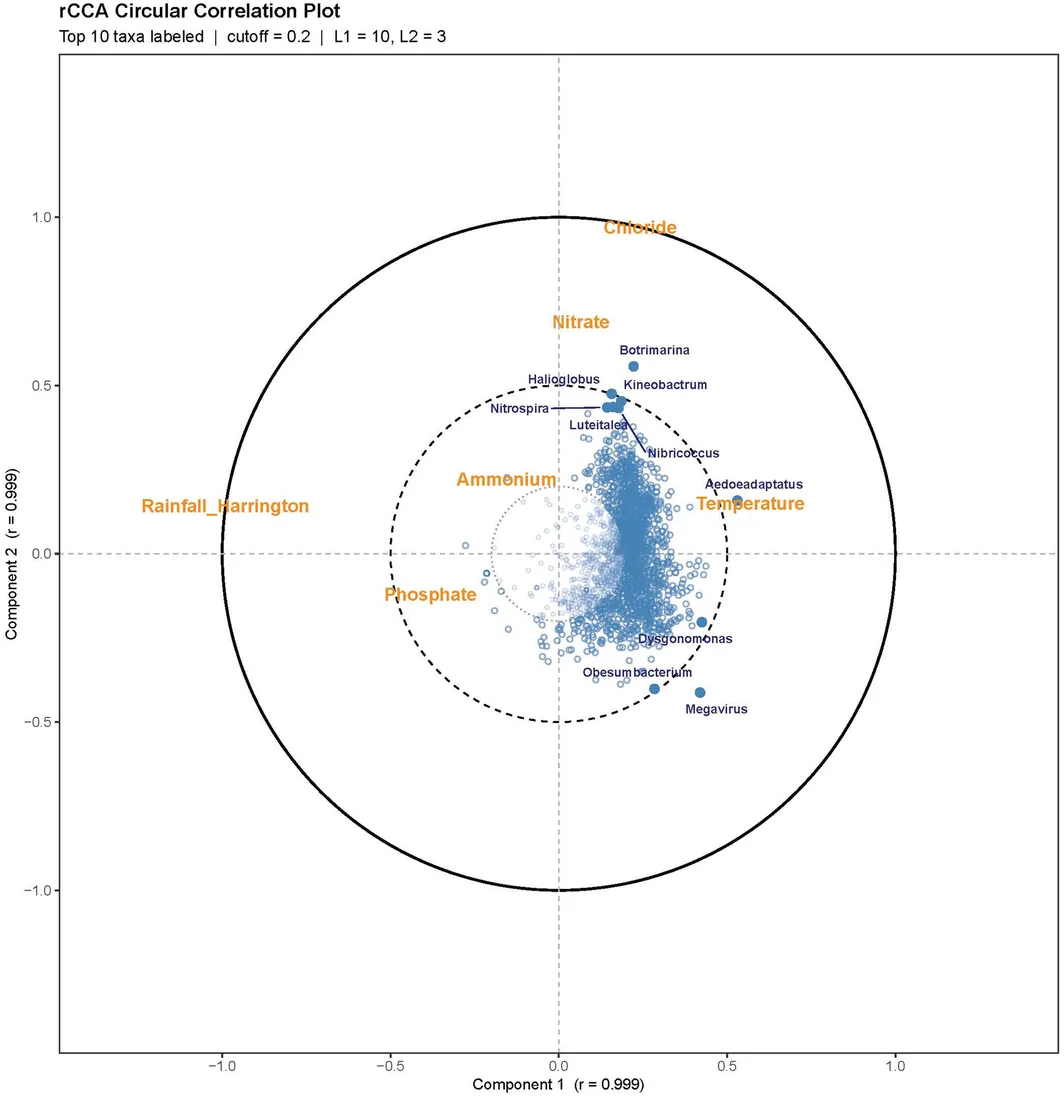
Regularized canonical correlation analysis (rCCA) of microbial communities and nutrients. Circular correlation plots showing the relationships between microbial taxa, both temperature and key nutrient concentrations (nitrate, chloride, phosphate, and ammonium). The plots visually represent the regularized canonical components. Variables near the outer circle have strong correlations with these components. Clusters of taxa indicate groups that exhibit similar associations with the measured environmental gradients.

Permutation testing (999 iterations) of the canonical cross‐loadings confirmed statistical significance for 10 of the 20 evaluated taxon‐component pairs. The analysis revealed a robust environmental gradient captured by Component 2 where six taxa such as *Botrimarina* (CL = 0.557, *p* < 0.001), *Halioglobus* (CL = 0.475, *p* = 0.004), *Kineobactrum* (CL = 0.453, *p* = 0.005), *Luteitalea* (CL = 0.436, *p* = 0.009), *Nitrospira* (CL = 0.435, *p* = 0.013), and *Nibricoccus* (CL = 0.433, *p* = 0.007) showed significant positive cross‐loadings exclusively on Component 2. A sensitivity analysis substituting the rainfall covariate confirmed that this Component 2 axis represents a highly reproducible, rainfall‐independent environmental gradient, maintaining significant associations for all six core taxa. However, while these specific genera showed robust statistical evidence of association with this nutrient gradient that exceeded chance expectations, their relative abundances were consistently below 0.1% (Table S7). Therefore, despite the mathematical strength of their statistical associations, these rare taxa likely have a highly limited functional impact on the bulk processes of the river ecosystem.

### 3.7 Functional Profiling

During functional profiling with HUMAnN3, an average of ~11% of reads across the dataset successfully mapped to the ChocoPhlAn and UniRef90 reference databases. The detailed per‐sample mapping statistics, which were evaluated to ensure sufficient hit rates for reliable pathway abundance estimation, are provided in Table S11.

We used two complementary bioinformatics methods, HUMAnN3 and MaAsLin2, to characterize the global patterns of functional potential of the riverine microbiome, and the relationship between environmental variables and pathways with relevance to biogeochemical cycling. PCoA based on Euclidean distances of pathway abundance profiles revealed that temporal factors—specifically month, season, and year—were significantly associated with microbial functional composition. In contrast, spatial differences among the four river sites were not statistically significant for functional pathway profiles (PERMANOVA, *R* ^2^ = 0.030, *p* = 0.55) (Figure 6d).

**FIGURE 6:**
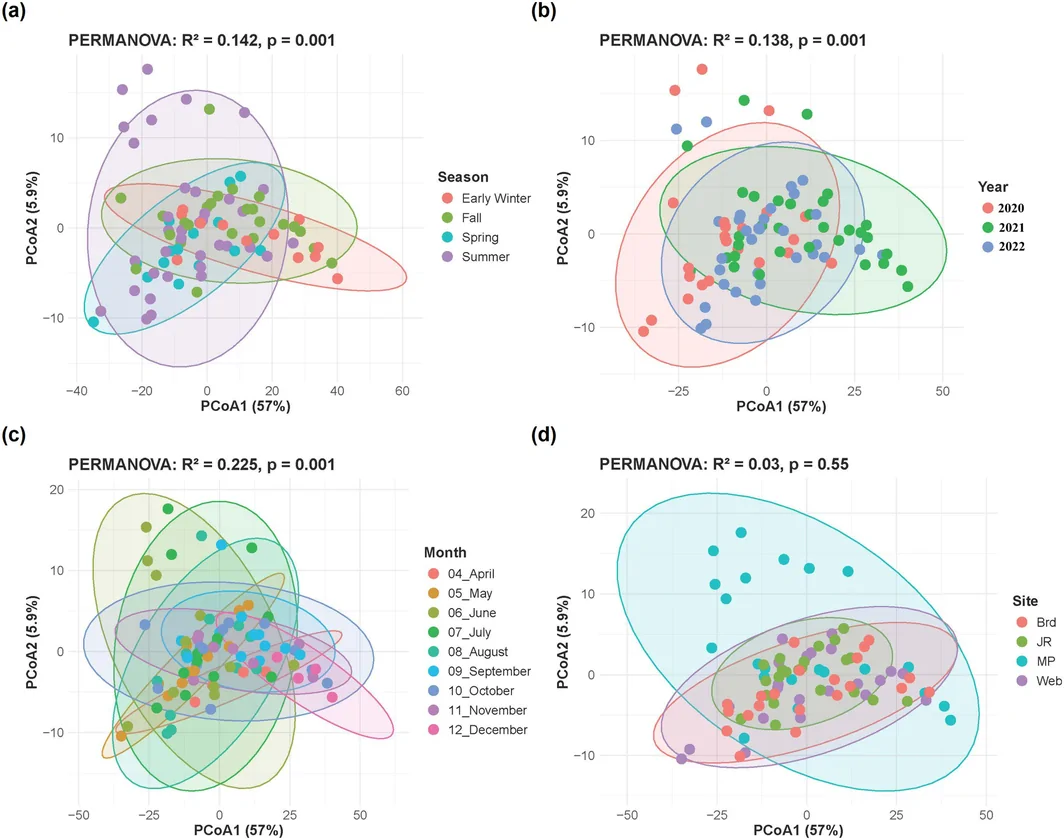
PCoA plots based on euclidean distance of functional pathway abundance profiles. Plots are derived from HUMAnN3 output and illustrate the clustering of pathway abundance profiles across season (a), year (b), month (c), and site (d). Points represent individual riverine metagenomes (*n* = 87), while ellipses indicate 95% confidence intervals for each group.

When samples were grouped by season, significant differences in functional pathway profiles were observed (PERMANOVA, *R* ^2^ = 0.142, *p* = 0.001) (Figure 6a), with clearer separation between summer and early winter samples, highlighting the potential role of seasonal cycles in shaping microbial pathways. Interannual variation was also significant for functional pathway profiles (PERMANOVA, *R* ^2^ = 0.138, *p* = 0.001) (Figure 6b), with distinct year‐specific profiles observed throughout the study period. Monthly variations explained the highest proportion of variance in functional pathway profiles (PERMANOVA, *R* ^2^ = 0.225, *p* = 0.001) (Figure 6c), indicating strong intra‐annual shifts.

These global patterns were further corroborated by MaAsLin2 differential abundance analysis. Pairwise seasonal comparisons revealed the sharpest contrasts between early winter and summer, with 265 and 284 pathways significantly different in abundance, respectively (Figure S6a). Early winter also differed markedly from spring (269 pathways), while the summer‐to‐fall transition showed a more moderate shift (114 pathways). In contrast, spring–summer (12–13 pathways) exhibited high functional redundancy. Yearly comparisons showed a pronounced separation between 2020 and 2021, with 240 pathways significantly different in abundance (Figure S6b). Interestingly, 2021 appeared as a functional outlier, differing significantly from both 2020 and 2022 (107 and 21 pathways, respectively), whereas 2021 and 2022 showed the highest similarity (only 8 and 5 pathways differing significantly).

Functional analysis identified a small spatial signature, with 17 unique pathways showing significant differential abundance across site comparisons. The Maple Plains (MP) site exhibited the most distinct functional profile; comparisons with MP as the reference yielded 7 significant pathways each when compared to Web and JR, and 3 when compared to Brd (Figure S6c). Conversely, comparisons between Web, JR, and Brd yielded negligible differences, suggesting that the spatial heterogeneity in functional potential is primarily driven by unique conditions at the MP site.

Regarding specific biogeochemical cycles, microbial pathways related to carbon, nitrogen, phosphorus, and sulfur showed distinct temporal oscillations (Figure S3a–d). Carbon fixation (e.g., reductive TCA) and photorespiration were most prominent during summer and fall, coinciding with peak primary productivity. These pathways significantly declined in early winter (log2 fold‐change −1.1 to −1.4, *q* ≤ 0.05; Table S8 and Figure S3a). Similarly, nitrogen cycling—including denitrification, nitrate reduction, and the urea cycle—was significantly less abundant in early winter compared to summer (log2 fold‐change −1.7 to −1.5, *q* ≤ 0.015; Table S8, Figure S3b).

The interannual analysis highlighted 2021 as a period of reduced metabolic potential. Relative to 2020 and 2022, 2021 showed significant decreases in methanol oxidation, carbon fixation (reductive TCA cycles I and II), and photorespiration. Nitrogen turnover followed this trend, with the urea cycle and denitrification pathways (Nitrate reduction I and VII) consistently less abundant in 2021 compared to other years (Table S9). Within the spatial analysis, the urea cycle was found in significantly higher relative abundance at JR compared to MP (log2 fold‐change = 1.42, q = 0.041; Table S10), representing the only major metabolic pathway shift detected between sites.

## 4 Discussion

In this study, our findings align with the primary hypothesis that temporal factors, specifically seasonal and inter‐annual differences, are key factors impacting the microbial community structure and functional potential in the Dunk River system. The observed temporal pattern may be a consequence of the unique hydrogeological setting on PEI, which contrasts with larger, more‐complex river systems studied elsewhere (Barberoux et al. 2025; Čačković et al. 2023; França et al. 2015; Land and Peters 2023; She et al. 2024). The island's underlying uniform, fractured bedrock aquifer provides a consistent, low‐microbial‐load source of groundwater that contributes to a more homogeneous microbial signal across the study sites, potentially outweighing the localized influence of land‐use activities. While the constant flow of cool groundwater limits microbial growth under normal conditions, the significant temporal shifts we observed, particularly the increased microbial activity in the warmer, drier year of 2020, highlight how short‐term changes in residence time and temperature can significantly alter these dynamics. The functional profiling also revealed a subtle spatial signature. While the overall signal remains influenced by PEI's unique hydrogeology, our results indicate that localized environmental pressures can still induce site‐specific functional shifts, particularly at the Maple Plains site.

### 4.1 Compositional and Diversity Dynamics

The microbial communities in the Dunk River were dominated by the phylum Pseudomonadota across all samples (68.43%), which is consistent with taxonomic baselines observed in freshwater lotic ecosystems worldwide (Blais et al. 2022; Hassell et al. 2018; Henson et al. 2018; A. Hu et al. 2017). Consistent with this phylum‐level dominance, the most abundant bacterial families identified were Comamonadaceae (20%), Flavobacteriaceae (10%), Pseudomonadaceae (5%), and Burkholderiaceae (4%), all from phylum Pseudomonadota. These core families are widely recognized for their roles in organic matter decomposition, nutrient cycling, and metabolic processing in rivers (Balmonte et al. 2016; Banerjee et al. 2018; Battin et al. 2008; Cai et al. 2013; Ding et al. 2023; Fang et al. 2023).

Although the dominant phyla are highly conserved across temperate freshwater networks, our family‐level profiles reveal distinct differences compared to other watersheds. For example, ecologically relevant freshwater riverine investigations in Atlantic catchments (Goldenberg‐Vilar et al. 2025) and the Yangtze River (Yao et al. 2024) both reported similar phylum‐level patterns but noted significant variation in the specific dominant families compared to our findings. Additional riverine metagenomic studies further support this concept by showing that while major phyla remain conserved, local environmental factors drive distinct shifts in the most abundant families (Reddington et al. 2020; Van Rossum and Rossum 2017).

At the genus level, *Flavobacterium* (9.32%) and *Rhodoferax* (3.82%) emerged as the most abundant taxa, exhibiting prominent increases during the dry summer months. These shifts suggest a rapid biological response to changing nutrient concentrations and low‐flow conditions. The high abundance and dynamic temporal shifts of *Flavobacterium* indicate that our water‐column samples capture an actively metabolizing community rather than dormant, passively transported cells. *Flavobacterium* species are highly efficient degraders of organic macromolecules in headwater streams (Battin et al. 2008), and their dominance likely reflects active growth on dissolved organic substrates derived from terrestrial runoff, potentially supplemented by natural dispersal from benthic biofilms (Cai et al. 2013). The strong temporal variations we observed in these groups peak during the 2020 drought and decline in subsequent years, showing a structured response to changing residence times and solute concentrations (Veach et al. 2016). Conversely, passive terrestrial transport is restricted to the low‐abundance “rare biosphere,” as illustrated by the tentative, low‐abundance detection (0.03%) of *Pseudarthrobacter*, which is a regional potato crop pathogen whose presence suggests a minor agricultural runoff signature in the water column.

This shift in community structure is further reflected in our diversity metrics. We observed sharp declines in Shannon diversity during the summer months, most notably during the 2020 drought. To ensure these trends were not artefacts of sequencing effort, we rarefied our count tables to a uniform depth, confirming that this decline represents a genuine biological reduction in community complexity. These drops in diversity likely reflect stronger deterministic environmental filtering and increased physical stress during low‐flow summer conditions.

While the distinct diversity patterns observed in 2020 initially suggested a general interannual effect, our pairwise post hoc tests revealed that the non‐drought years (2021 and 2022) were statistically indistinguishable. This indicates that the global year effect does not reflect consistent, year‐to‐year community turnover. Instead, the distinctiveness of the 2020 community represents a temporary response to the extreme low‐rainfall conditions of that year. Interestingly, our raw water chemistry data (Table S4) shows that solute concentrations, such as chloride and nitrate, did not significantly increase or concentrate during the 2020 drought compared to subsequent years. Instead, this distinctiveness was likely driven by the physical and hydrological consequences of the drought, specifically extremely low streamflows, prolonged water residence times (Table S2), and significantly elevated midsummer water temperatures (Table S3). Rather than a direct chemical concentration effect, the 2020 interannual variation is best understood as a temporary shift in community structure driven by drought‐induced hydrological stabilization and increased water temperature (Crump et al. 2026; Mahmood et al. 2024; Paradis et al. 2018; Ren et al. 2019).

While our results indicate that temporal factors are the primary identifiable correlates of microbial community structure among the variables measured, the modest effect sizes indicate that a substantial majority of the community variance remains unexplained. Standard physicochemical variables such as pH, dissolved oxygen (DO), and dissolved organic carbon (DOC) were not measured in this study due to logistical constraints. However, dissolved oxygen hourly swings can exceed monthly grab samples, meaning periodic grab sampling is often an ineffective predictor of biological dynamics (M. Coffin et al. 2021). Previous evaluations of PEI aquatic systems have demonstrated that even when a comprehensive suite of these standard chemical variables is measured, they often fail to predict or correlate strongly with macroinvertebrate and aquatic plant community structure (Knysh et al. 2016). Similarly, primary production indicators in these shallow, well‐mixed systems often resist explanation by basic physical or nutrient covariates (Coffin et al. 2018).

Therefore, the high unexplained variance observed here is likely a characteristic feature of riverine microbial ecology, reflecting stochastic assembly processes and local internal nutrient cycling rather than simply the omission of bulk water‐chemistry parameters. In open and hydrologically dynamic river systems, it is standard for primary temporal or environmental variables to explain only a minor fraction of total variance (Kodera et al. 2023). Because lotic ecosystems are continuously seeded by allochthonous sources such as soil runoff, tributaries, and atmospheric deposition, stochastic dispersal can frequently outweigh deterministic environmental filtering (Ren et al. 2019). Ultimately, while the seasonal and interannual shifts detected here represent biologically meaningful responses, high residual variance remains a characteristic baseline feature of complex riverine ecosystems like showcased in the Lower Mississippi River Study (Payne et al. 2020).

### 4.2 Metabolic Functional Potential and Seasonal Dynamics

Functional metagenomic profiling also showed a stronger association with temporal variations than with specific sites. This pattern is consistent with recent research on freshwater and coastal ecosystems (Barberoux et al. 2025; Xie et al. 2025). Our findings align with this by showing that even in a temperate river, the distinct seasonal conditions and resulting changes in temperature and hydrology likely impose a stronger selective pressure than the relatively homogenous conditions between our sampling sites. We found significant enrichment of pathways associated with carbon, nitrogen, phosphorus, and sulfur cycling during the summer months and in 2020 relative to colder seasons and subsequent years. Specifically, the strong summer enrichment across carbon fixation, methyl‐phosphonate degradation, nitrogen turnover, phosphorus degradation, and sulfur assimilation pathways likely reflects conditions of higher microbial productivity (Bier et al. 2015; Crump et al. 2012). Warmer temperatures, longer photoperiods, and enhanced primary production in summer increase metabolic rates and substrate availability, a pattern previously observed in agriculturally impacted watersheds (Cross et al. 2022; Griffiths et al. 2013). The interannual differences highlight that microbial function was consistently greater in 2020 compared to subsequent years, with 2021 showing the most pronounced reductions. This suggests that environmental conditions specific to 2020 may have favoured a higher apparent microbial metabolic capacity, possibly due to interannual variation in precipitation. The lower precipitation in 2020 could have led to reduced water volume and warmer temperatures, thereby concentrating available substrates and increasing the metabolic rate of the microbes present. This interannual variability of the riverine microbiome underscores the importance of multi‐year monitoring to distinguish between natural oscillations and potential anthropogenic impacts.

### 4.3 Subtle Spatial Differences Across Sampling Sites

We found that while spatial differences account for a small portion of the total variation, the functional potential is not entirely uniform across the watershed. The analysis identified not many unique pathways with significant spatial differential abundance, primarily centered on the Maple Plains (MP) site. This site‐specific signature likely reflects localized land‐use impacts or micro‐habitat variations that are not captured by the broad taxonomic profiles.

Specifically, the significantly higher relative abundance of the urea cycle at the Johnston River (JR) site compared to MP (log_2_ fold‐change = 1.42) provides an example of localized differences in functional potential. JR exhibits moderate nitrate levels despite low chloride and phosphate concentrations, suggesting a unique nitrogen processing dynamic. This enrichment in urea‐related pathways represents the only major metabolic pathway shift detected between sites (Table S10). It likely reflects localized inputs of organic nitrogen from agricultural activities in this sub‐watershed, where organic nitrogen sources such as animal manures or crop residues are frequently utilized (Zebarth et al. 2015).

Consistent with the lack of statistical significance for the site variable in our taxonomic profiling (PERMANOVA, *R* ^2^ = 0.046, *p* = 0.072), the substantial overlap of samples among different river locations in our PCoA plots (Figure 4) indicates that spatial separation did not lead to major differences in community structure. The lack of a statistically significant spatial effect on taxonomic composition should be interpreted cautiously, as the unbalanced seasonal sampling design may leave the study underpowered to detect moderate spatial differences. Nevertheless, the small calculated effect size suggests that if spatial structuring occurs, it is likely a weaker driver of community composition compared to temporal factors. This limited spatial differentiation is frequently observed in small temperate river networks (Akinwole et al. 2021) and is consistent with the underlying hydrogeology of the study area.

The island is underlain by a relatively uniform, fractured bedrock aquifer (Allan Freeze and Cherry 1979; Van De Poll 1988) which discharges a consistent, low‐microbial‐load source of cool groundwater into the streams. This predominant hydrological influence may outweigh localized effects from agricultural activity on the overall microbial community structure, contributing to a more uniform microbial signal across sites (Jiang et al. 2015; Jiang and Somers 2009). The unique hydrogeology, characterized by a small landmass, short stream lengths, and groundwater‐fed streams, plays a crucial role, as the short water residence time limits the time available for bacterial growth (Stegen et al. 2016). However, in drier years like 2020, reduced groundwater recharge can slow water movement and raise stream temperatures (Rivard, Michaud, Deblonde, et al. 2008; Rivard, Michaud, Lefebvre, et al. 2008) allowing for increased bacterial abundance. This confluence of uniform geology and unique hydrology creates a distinctive microbial dynamic, which may not be comparable to that of larger, more complex river systems with different inputs and stream order, such as the Mississippi River where species sorting plays a more important role (Henson et al. 2018). Similar observations have been made in other smaller systems, such as the Willamette basin in Oregon (Land and Peters 2023), and a karstic aquifer system in Portugal, where hydrogeochemical conditions significantly influenced community composition, further supporting the idea that local hydrology is a key driver in these smaller, groundwater‐dependent streams (França et al. 2015).

This lack of noticeable spatial differentiation can also be explained by the concept of homogenizing dispersal or mass effects, although it is likely a minor factor in our specific system given that our sites are not situated along a direct downstream gradient (Mouquet and Loreau, n.d., 2002, 2003b, 2003a). In this context, dispersal could occur through a variety of mechanisms, including continuous groundwater discharge or a dense network of sub‐surface flow paths connecting the sites. This constant movement of cells can potentially dampen the influence of localized environmental selective pressures, leading to a more homogenous community structure across locations. In essence, the sheer volume of migrating cells, representing a “mass effect”, can override the “species sorting” that would typically lead to distinct, environmentally filtered communities (Cadotte 2006; Mouquet and Loreau 2002; Shanafelt et al. 2015). This finding is supported by other research on riverine microbial ecology. For example, a study on the Elwha River (Pan et al. 2022) highlighted how high hydrological connectivity can promote microbial community coalescence in riverine floodplains, while a study of the Chishui River (Wu et al. 2023) explored how homogeneous selection and mass effects can dominate community assembly even along strong environmental gradients.

Our initial sampling design deliberately selected sites across a gradient of agricultural intensity, ranging from 60% to 96% land cover (Table 1), to capture localized anthropogenic pressures. However, the resulting spatial homogeneity indicates that the continuous influx of groundwater overrides these localized agricultural gradients. Because the streams are continuously flushed by the same uniform aquifer (Jiang et al. 2015), this massive and geochemically stable water source physically buffers the river's microbiome against local runoff disturbances. When transient localized intrusions occur, such as agricultural runoff differing by sub‐catchment, the constant baseflow rapidly drives the microbial community back to a shared equilibrium state (Wang et al. 2025). Consequently, the river ecosystem buffers itself against site‐specific anthropogenic inputs, preventing the establishment of distinct spatial microbial signatures based on local farming intensity.

Lastly, our initial global analysis showed no clear relationship between the bulk of the microbial community composition and the measured nutrient concentrations. This absence of global correlations likely reflects the stochastic disturbance of high‐flow seasons, which rapidly transport allochthonous taxa and continuously restructure the microbial community (Chen et al. 2024). From a resilience perspective, these transient hydrological states prevent stable community organization. PEI streams are uniquely suited to test this, as groundwater contributes nearly 100% of stream baseflow during the summer (Mahmood et al. 2024). High summer evapotranspiration halts surface runoff, isolating the system from transient terrestrial inputs (Paradis et al. 2018). In the Dunk River watershed, where approximately 80% of the land is agricultural, this groundwater is the primary transport mechanism for agricultural nitrate entering the streams (Crossley et al. 2025). This low‐flow period initiates microbial community succession; when streamflow slows down during the dry summer, the water spends more time in the channel, giving resident microbes enough time to grow and adapt to the local environment rather than being quickly washed downstream (Crump et al. 2026). Within this stabilized ecological state, deterministic niche selection becomes more prominent.

Consistent with this hypothesis, our exploratory summer rCCA identified associations between a small number of low‐abundance genera and nutrient levels (Figure 5, Figure S5). While the initial rCCA identified weak associations between certain low‐abundance genera and nutrient levels, rigorous permutation testing revealed that only a subset of these relationships, specifically those involving nitrate, chloride, and temperature, were statistically robust. Because these taxa are present at relative abundances below 0.1%, they are theoretically vulnerable to false‐positive classification stemming from k‐mer matching artefacts. However, our permutation testing generated explicit confidence intervals demonstrating that 10 of these specific taxon‐component associations highly exceed what would be expected by random chance. Specifically, six core taxa, including *Botrimarina*, *Halioglobus*, and the nitrite‐oxidizer *Nitrospira*, formed a highly reproducible environmental gradient on Component 2 that remained robust during sensitivity testing with alternate rainfall covariates. The coherent directionality and structural stability of this nutrient‐driven component make a random bioinformatics artefact less plausible, suggesting these taxa represent genuine ecological responses to localized nutrient availability. Associations with phosphate and ammonium failed to reach significance, indicating that signals from extremely rare taxa, such as the viral genus *Megavirus*, in these specific gradients were likely classification artefacts or stochastic noise. This highlights the necessity of statistical validation when interpreting the ecological relevance of rare taxa. Nevertheless, we interpret their biological role with caution. While rare taxa can exhibit a robust relationship with the environment, their extremely low abundance suggests a limited functional impact on the bulk biogeochemical processes of the river ecosystem compared to the dominant community members.

### 4.4 Limitations

Several limitations of the current study should be acknowledged. First, the association between low‐abundance taxa and nutrients may be sensitive to sequencing artefacts and effects of compositionality, a common challenge in metagenomic studies where relative abundance data can mask true changes in absolute abundance. While methods like Aitchison distance can help mitigate these effects, more robust solutions often require absolute quantification via techniques such as quantitative PCR (qPCR) or the use of internal spike‐in standards (Crossette et al. 2021; Gloor et al. 2016; Pereira‐Marques et al. 2019). (Crossette et al. 2021; Gloor et al. 2016; Pereira‐Marques et al. 2019).

Second, the use of the Illumina MiSeq platform (300 bp × 2), while providing the advantage of longer read lengths that improve taxonomic and functional alignment accuracy, resulted in a lower overall sequencing throughput compared to high‐capacity platforms. While the sequencing depth in this study (mean 7.19 million reads; range 1.91 to 30.44 million reads) is lower than what is typically required for high‐resolution genome reconstruction, it aligns with shallow shotgun metagenomics approaches utilized for comparative ecological profiling. This represents a trade‐off in study design, prioritizing extensive spatiotemporal sample breadth (*n* = 87 across four sites over 3 years) over deep per‐sample throughput to achieve the statistical power necessary for evaluating multi‐year watershed dynamics. Methodological evaluations demonstrate that taxonomic and functional community structures can be reliably characterized at shallow sequencing depths, including those below one million reads per sample (Hillmann et al. 2018). Benchmarking in riverine environments specifically indicates that a depth of one million reads yields stable taxonomic profiles (Gweon et al. 2019). Furthermore, to mitigate comparability issues introduced by sequencing depth heterogeneity across our sample set, downstream alpha‐diversity and comparative analyses utilized rarefied and compositionally normalized data. Because our primary objectives focused on broad community structure rather than strain‐level resolution, this depth, combined with appropriate statistical handling, provides adequate analytical resolution to assess spatial and temporal community dynamics. Nevertheless, this lower depth likely limited our ability to detect rare, low‐abundance functional pathways and construct complete metagenome‐assembled genomes (MAGs).

Third, environmental freshwater microbiomes are notoriously poorly represented in standard reference databases compared to clinical environments. Consequently, a portion of the metagenomic reads remained unmapped during the HUMAnN3 functional profiling. While the mapping rates were sufficient to capture core biogeochemical cycling functions, this incomplete database representation may limit our ability to detect rare, novel, or highly specialized metabolic pathways, meaning our functional profiles primarily reflect the well‐characterized fraction of the lotic microbial community (Pérez‐Cobas et al. 2020). Furthermore, these taxonomic and functional annotations infer potential roles in nutrient cycling but do not confirm activities, as gene expression and enzymatic function were not directly measured (Rodríguez‐Ramos et al. 2023).

Fourth, our size‐fractionation methodology specifically targeted the free‐living bacterioplankton community. Because headwater environments are heavily influenced by benthic biofilms, which are major drivers of lotic ecosystem functioning, our functional profiles represent only the water‐column fraction and only minimally capture the metabolic contributions of biofilm‐associated microbiomes. Finally, spatial and temporal resolution were limited, potentially obscuring short‐term microbial responses to environmental perturbations (Stach et al. 2025). As noted previously, unmeasured factors such as pH, dissolved oxygen (DO), dissolved organic carbon, salinity, and turbidity also likely contributed to the unexplained variance in the observed community dynamics (Konopka et al. 2015).

### 4.5 Conclusions and Future Work

Our study suggests that temporal dynamics, particularly seasonal and interannual shifts, are significant drivers of both microbial community composition and functional potential in this river system. While taxonomic composition was largely homogeneous across sites, the functional profiling revealed subtle but significant spatial signatures, particularly at the Maple Plains site. This highlights that functional metagenomics might be a more sensitive choice of characterization tool than taxonomic profiling for detecting site‐specific ecological responses in groundwater‐dominated systems. While this study identified statistically detectable seasonal and temporal shifts in the microbial community, these factors explained a limited proportion of the total community variation. Among the measured environmental parameters, temporal factors were the primary identifiable drivers of community structure; however, a substantial portion of the variance remained unexplained. This high residual variance suggests that stochastic assembly processes, continuous hydrological mixing, and unmeasured ecological variables account for the majority of the community dynamics in this system.

These findings align with a growing body of literature that highlights the dominant role of time‐based factors in shaping riverine ecosystems, which suggests that future work should aim to pinpoint the specific environmental variables—such as short‐term spikes in temperature or nutrient concentrations—that directly correlate with and drive changes in the microbial community. Furthermore, coupling microbial data with hydrological and geochemical modelling could enhance our understanding of how these communities respond to environmental disturbances and to what extent they are governed by local selection versus homogenizing dispersal. A more comprehensive approach would integrate high‐resolution temporal sampling with functional validation techniques like meta‐transcriptomics to confirm the activity of key nutrient‐cycling pathways (Belnap et al. 2005; Hauptfeld et al. 2024).

To build upon these findings, future investigations should combine deeper sequencing with long‐read technologies. Increased sequencing depth would provide more comprehensive and accurate assessments of microbial diversity and improve the coverage required for functional profiling tools such as HUMAnN3 to infer complete or near‐complete metabolic pathways, particularly among lower‐abundance taxa. In parallel, long‐read sequencing could overcome assembly bottlenecks of short‐read approaches, enabling high‐quality reconstruction of MAGs and more robust linkages between specific microbial lineages and their biogeochemical functions. Furthermore, because the current sampling design focused exclusively on the free‐living microbial fraction, future studies should incorporate paired sampling of epilithic and benthic biofilms. Comparative analysis of the water‐column and biofilm‐associated microbial communities will be critical for resolving the spatial partitioning of biogeochemical processes and terrestrial organic matter transformation within these headwater ecosystems.

## Author Contributions

**Sneha Surya Narayana Murthy:** methodology, formal analysis, investigation, writing – original draft, writing – review and editing, visualization. **Jiacheng Chuan:** methodology, software, data curation, writing – review and editing. **Yefang Jiang:** data curation, methodology, writing – review and editing, conceptualization, funding acquisition, resources. **Wen Chen:** methodology, validation, writing – review and editing. **Robert G. Beiko:** conceptualization, methodology, validation, funding acquisition, supervision, resources, writing – review and editing. **Xiang Li:** conceptualization, funding acquisition, methodology, data curation, supervision, resources, writing – review and editing, validation.

## Funding

This work was supported by the Canadian Food Inspection Agency, Natural Sciences and Engineering Research Council of Canada, Dalhousie University.

## Conflicts of Interest

The authors declare no conflicts of interest.

## Supporting information


**Figure S1:** (a). Distribution of sequence quality metrics across all samples. Histograms illustrate the distribution of (top left) percent duplicates, (top right) GC content, (bottom left) average sequence length, and (bottom right) total sequences (in millions of reads) across all metagenomic samples. (b) Summary of metagenomic sample distribution across year, site, season, and month. Bar plots display the number of river metagenome samples collected from the four sites categorized by (a) year, (b) site, (c) season, and (d) month.
**Figure S2:** Relative abundance of bacterial communities across four river sites at the (a) Family and (b) Species levels. Coloured bars represent dominant taxa, with less abundant groups with a raw count of less than 10 reads collapsed as “Others.”
**Figure S3:** Heatmaps showing the relative abundance of selected metabolic pathways across riverine metagenome samples. (a) carbon fixation and photorespiration‐related functions; (b) nitrogen metabolism, including nitrate reduction, denitrification, and the urea cycle; (c) phosphorus degradation pathways; and (d) sulfur metabolism pathways. Samples are annotated by year, site, and season in the top bar, while rows correspond to pathways and columns to samples. Data are log‐transformed normalized abundances, with warmer colours (red) indicating higher and cooler colours (blue) indicating lower relative abundance. Hierarchical clustering of rows groups pathways with similar abundance patterns across spatial and temporal gradients.
**Figure S4:** The rCCA circular correlation plots, which illustrate the relationships between nutrient factors, temperature and rainfall (orange labels) and microbial genus profiles (blue points), across all four river sites and 3 years. The two circles represent correlation thresholds, with points near the outer circle indicating stronger correlations. Taxa clustering near the center represents weak or insignificant correlations with the environmental variables.
**Figure S5:** Heatmap from rCCA showing top 10 correlations between microbial genera and environmental (Env) variables. Rows (genera) and columns (nutrients, temperature, rainfall) are hierarchically clustered based on their correlation profiles, with colour intensity indicating the strength and direction of associations (Table S13).
**Figure S6:** Directional MaAsLin2 pairwise comparisons. The seasonal comparison plot (a) shows the number of significantly different pathways between seasonal pairs, with the rows as the reference category. These pathways were identified as significant using p‐adjusted values calculated by the Benjamini–Hochberg method, with an FDR threshold of 0.25, and a significance cutoff of *p* < 0.05 (Tables S14 and S15). The yearly comparison plot (b) similarly shows the number of significant pathways between yearly pairs. The site comparison plot (c) shows the number of significant pathways between site pairs.

**Table S1:** Quality‐control statistics derived from the processed metagenomic dataset.
**Table S2:** Precipitation amounts and summary statistics for two locations in Prince Edward Island near the study site.
**Table S3:** Temperature in degrees Celsius for the four sampled streams across the 3 years of the study.
**Table S4:** Monthly nutrient concentrations for the four sampled streams across the 3 years of the study.
**Table S5:** Diversity metrics richness and shannon index—Genus Level.
**Table S6:** Diversity metrics richness and shannon index—Species Level.
**Table S7:** Relative abundance of inferred genera across all metagenomic samples.
**Table S8:** MaAsLin2 differential abundance analysis—Season‐wise Comparison.
**Table S9:** MaAsLin2 differential abundance analysis—Year‐wise Comparison.
**Table S10:** MaAsLin2 differential abundance analysis—Site‐wise Comparison.
**Table S11:** Per‐sample HUMAnN3 functional profiling mapping rates for all 87 river metagenome samples, including nucleotide (ChocoPhlAn/Bowtie2) and translated (UniRef90/Diamond) alignment statistics, with summary statistics across all samples.

## Data Availability

The datasets generated from the next‐generation sequencing in this study are available in the NCBI Sequence Read Archive (SRA) repository, Bioproject ID: PRJNA1416788.
