# Supplementary material for: Seasonal and Environmental Influences on Microbial Community Dynamics in the Dunk River Watershed, Prince Edward Island, Canada
Source: Environ Microbiol. 2026 Nov 2;28(11):e70469. doi: 10.1111/1462-2920.70469 (PMC13727502; doi:10.1111/1462-2920.70469)
Supplement: Supplementary file 1 [file EMI-28-e70469-s002.docx]

# SI Figures for Manuscript

**Figure S1 (a)**. **Distribution of sequence quality metrics across all samples.** Histograms illustrate the distribution of (top left) percent duplicates, (top right) GC content, (bottom left) average sequence length, and (bottom right) total sequences (in millions of reads) across all metagenomic samples.

(b) **Summary of metagenomic sample distribution across year, site, season, and month**. Bar plots display the number of river metagenome samples collected from the four sites categorized by (a) year, (b) site, (c) season, and (d) month.

**Figure S2**. Relative abundance of bacterial communities across four river sites at the (a) Family and (b) Species levels. Colored bars represent dominant taxa, with less abundant groups with a raw count of less than 10 reads collapsed as “Others.”

**Figure S3**. Heatmaps showing the relative abundance of selected metabolic pathways across riverine metagenome samples. (a) carbon fixation and photorespiration-related functions; (b) nitrogen metabolism, including nitrate reduction, denitrification, and the urea cycle; (c) phosphorus degradation pathways; and (d) sulfur metabolism pathways. Samples are annotated by year, site, and season in the top bar, while rows correspond to pathways and columns to samples. Data are log-transformed normalized abundances, with warmer colors (red) indicating higher and cooler colors (blue) indicating lower relative abundance. Hierarchical clustering of rows groups pathways with similar abundance patterns across spatial and temporal gradients.

**Figure S4.** The rCCA circular correlation plots, which illustrate the relationships between nutrient factors, temperature and rainfall (orange labels) and microbial genus profiles (blue points), across all four river sites and three years. The two circles represent correlation thresholds, with points near the outer circle indicating stronger correlations. Taxa clustering near the center have weak or insignificant correlations with the environmental variables.

**Figure S5**. Heatmap from rCCA showing top 10 correlations between microbial genera and environmental (Env) variables. Rows (genera) and columns (nutrients, temperature, rainfall) are hierarchically clustered based on their correlation profiles, with color intensity indicating the strength and direction of associations. (**Table S13**)

**Figure S6**: **Directional MaAsLin2 pairwise comparisons.** The seasonal comparison plot (a) shows the number of significantly different pathways between seasonal pairs, with the rows as the reference category. These pathways were identified as significant using p-adjusted values calculated by the Benjamini–Hochberg method, with an FDR threshold of 0.25, and a significance cutoff of *p* < 0.05 (**Table S14, S15**). The yearly comparison plot (b) similarly shows the number of significant pathways between yearly pairs. The site comparison plot (c) shows the number of significant pathways between site pairs.
